# Multifunctional Bioactivity of *Saccharomyces cerevisiae* Extracellular Vesicle in Hair Follicle-Related Cellular Models

**DOI:** 10.3390/molecules31071171

**Published:** 2026-04-01

**Authors:** Hannah S. Park, Eunji Shin, Sehyun Shin

**Affiliations:** 1 Engineering Research Center for Biofluid Biopsy, Seoul 02841, Republic of Korea; 2 School of Mechanical Engineering, Korea University, Seoul 02841, Republic of Korea

**Keywords:** extracellular vesicles, *Saccharomyces cerevisiae*, hair follicle, dermal papilla cells, microbial EVs, anti-inflammatory activity, antioxidant activity

## Abstract

Extracellular vesicles (EVs) derived from microbial sources, including beer yeast (*Saccharomyces cerevisiae*), have recently attracted increasing attention as bioactive nanostructures with potential biomedical and cosmetic applications. In this study, EVs were isolated from Saccharomyces cerevisiae (beer yeast) using an electrokinetic ion-binding filtration system, followed by tangential flow filtration (TFF)-based buffer exchange. Their physicochemical characteristics and hair follicle-related biological activities were systematically evaluated. Nanoparticle tracking analysis demonstrated a mean particle size within the typical EV range, and zeta potential analysis confirmed a negatively charged surface. Cryo-transmission electron microscopy further verified the presence of lipid bilayer-enclosed nanovesicles. Biological activity was assessed in human dermal papilla cells, keratinocytes, and dermal fibroblasts, which collectively represent key components of the hair follicle microenvironment. At non-cytotoxic concentrations, yeast-derived EVs enhanced dermal papilla cell proliferation and promoted keratinocyte migration. The EVs attenuated pro-inflammatory cytokine expression under stimulated conditions and upregulated collagen-related gene expression in dermal fibroblasts. In addition, measurable antioxidant activity was observed. Collectively, these findings indicate that *S. cerevisiae*-derived extracellular vesicles exhibit multifunctional bioactivity relevant to the regulation of hair follicle-associated cellular processes. This study supports the potential of microbial EVs as scalable bioactive platforms for modulating hair follicle microenvironmental homeostasis.

## 1. Introduction

Extracellular vesicles (EVs) are nano-sized membrane-bound structures secreted by cells that carry diverse biomolecules, including proteins, lipids, and nucleic acids, and they play important roles in intercellular communication, immune regulation, and tissue regeneration [1]. As EV research has rapidly expanded in recent years, the need for standardization in isolation, characterization, and functional validation has become increasingly recognized. The International Society for Extracellular Vesicles (ISEV) has addressed this need through the MISEV guidelines, which provide criteria for EV definition and analysis to improve reproducibility and reliability in the field [2]. Within this framework, EVs are now regarded not merely as cellular byproducts but as functional biological delivery platforms.

To date, most EV studies have focused on vesicles derived from mammalian cells. However, increasing evidence shows that fungi and yeast also secrete nano-sized vesicular structures, leading to growing interest in fungal EV biology [3]. Fungal-derived vesicles have been reported to contain proteins, RNA, lipids, and other biomolecules, and to participate in host–pathogen interactions, immune modulation, and intercellular signaling [4,5]. Nevertheless, classical EV marker proteins and biogenesis pathways established in mammalian systems are not always directly applicable to fungal vesicles. For this reason, some studies have referred to fungal vesicles as vesicle-like nanoparticles or EV-like particles rather than extracellular vesicles [4,6]. Despite this nomenclature issue, the presence of membrane-bound morphology, nanoscale size distribution, secretion mechanisms, and biologically active cargo has led many recent studies to classify fungal vesicles within the EV category [3,4,5]. In accordance with this current research perspective, the vesicular structures isolated from Saccharomyces cerevisiae in the present study are referred to as extracellular vesicles (EVs).

The yeast Saccharomyces cerevisiae is a well-established biological resource with a long history of safe use in food and industrial applications. Recently, EVs derived from this organism have been proposed as potential functional biological delivery systems [5,7]. Previous work on fungal vesicles has largely focused on their immunological roles in pathogenic contexts, whereas their physiological functions in non-infectious environments and their potential industrial applications—particularly in relation to skin and hair follicle biology—remain relatively underexplored [3,5]. Therefore, studies that simultaneously validate both the physicochemical properties and biological functions of yeast EVs are needed.

Skin and hair follicle tissues maintain homeostasis through complex and interconnected processes involving cell proliferation, cell migration, inflammatory regulation, oxidative stress defense, and extracellular matrix (ECM) remodeling [8]. A growing number of EV studies have shown that EVs can simultaneously promote keratinocyte migration, enhance fibroblast collagen synthesis, suppress inflammatory cytokine responses, and reduce oxidative stress, suggesting their multifaceted role in skin regeneration [9]. In hair follicle biology, proliferation and survival of dermal papilla cells are recognized as key determinants of hair growth regulation, and EVs have been proposed as potential biological regulators capable of modulating these cellular processes [10].

In current hair growth research, minoxidil is widely used as a standard positive control for promoting follicular cell proliferation [11] and serves as a benchmark for evaluating the efficacy of new bioactive materials. However, while minoxidil primarily acts through vascular and signaling pathway-dependent mechanisms, EVs represent multifunctional platforms capable of delivering multiple biomolecular signals simultaneously. Consequently, EV-based materials are increasingly considered as integrated regenerative regulators that may influence not only cell proliferation but also inflammatory modulation, oxidative stress protection, and ECM synthesis in parallel [9,10].

In this study, the term extracellular vesicles (EVs) is used to describe nanoscale membrane-bound particles isolated from Saccharomyces cerevisiae, based on their morphology and physicochemical characteristics, including size distribution, surface charge, and vesicular structure. We acknowledge that comprehensive molecular characterization (e.g., EV-specific markers) was not performed; therefore, the current classification is based on physicochemical evidence consistent with previous reports on yeast-derived vesicles.

The particles were isolated using a combined strategy involving pre-filtration, charge-based capture, and subsequent concentration steps, followed by comprehensive physicochemical characterization. Hereafter, they are referred to as yeast-derived EVs (yeast EVs). Using human dermal papilla cells, keratinocytes, and fibroblast models, we evaluated the effects of these EVs on cell viability, migration, inflammatory response, antioxidant activity, and collagen synthesis. This study aims to demonstrate that yeast EVs may function as multifunctional biological platforms contributing to the regulation of skin and hair follicle homeostasis.

## 2. Results

**Figure 1:**
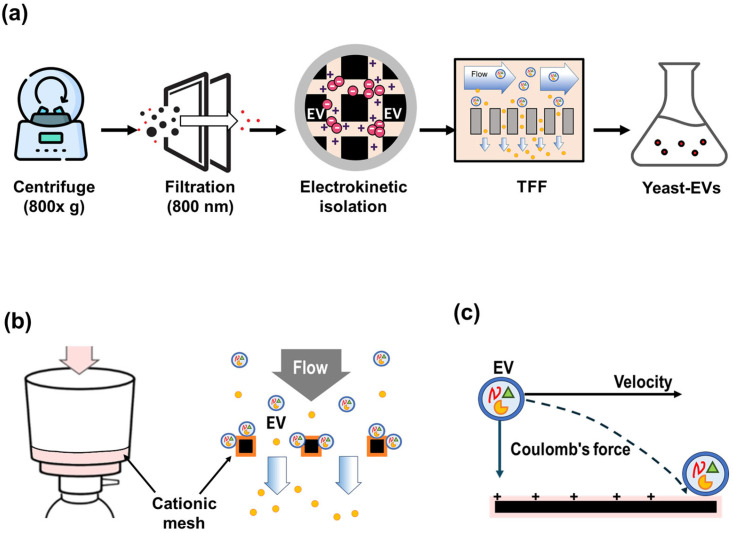
Isolation and electrokinetic purification strategy for Saccharomyces cerevisiae-derived extracellular vesicles (EVs). (**a**) Schematic overview of the EV isolation workflow, including low-speed centrifugation for removal of cellular debris, 800 nm filtration, electrokinetic charge-based capture using a cationic mesh (ExoFilter), and subsequent tangential flow filtration (TFF) for buffer exchange and concentration. (**b**) Conceptual illustration of charge-mediated EV capture, where negatively charged yeast EVs selectively bind to the positively charged mesh surface under flow conditions while soluble impurities pass through. (**c**) Electrokinetic binding mechanism showing Coulombic interaction between EV membranes and the cationic matrix, enabling selective retention followed by elution and downstream concentration.

To evaluate the biological effects of Saccharomyces cerevisiae-derived extracellular vesicles (EVs), vesicles were first isolated using the electrokinetic filtration workflow illustrated in Figure 1. The isolated vesicles were subsequently characterized to confirm their structural and physicochemical properties before proceeding to functional assays.

### 2.1. Isolation and Physicochemical Characterization of Yeast EVs

**Figure 2:**
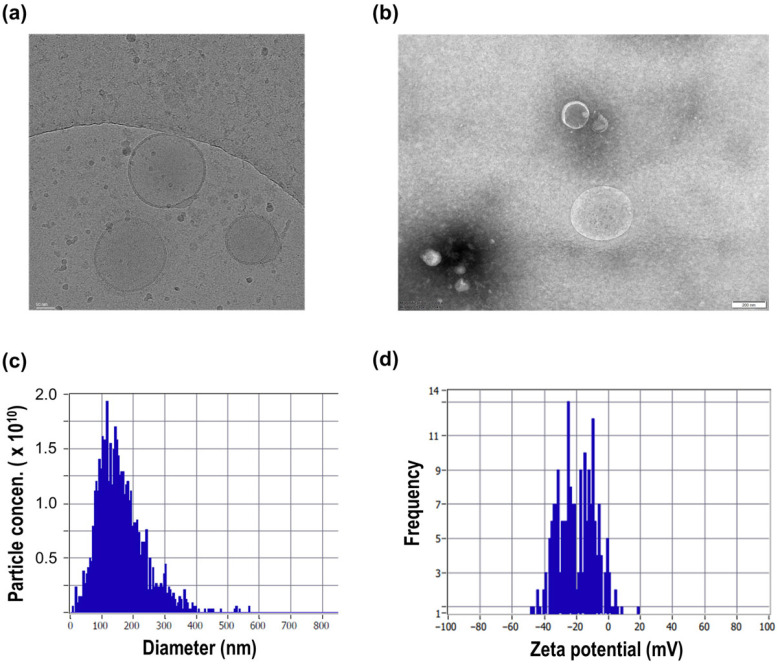
Physicochemical characterization of Saccharomyces cerevisiae-derived extracellular vesicles. (**a**) Cryo-TEM image showing intact vesicular morphology with well-defined membrane structures. (**b**) TEM image confirming the spherical shape and structural integrity of the isolated vesicles. (**c**) Nanoparticle tracking analysis (NTA) illustrating the size distribution of the EV population. (**d**) Zeta potential distribution demonstrating the negatively charged surface of yeast EVs.

The morphology of the isolated vesicles was first examined using cryogenic transmission electron microscopy (cryo-TEM) and conventional transmission electron microscopy (TEM). Cryo-TEM images revealed round, membrane-enclosed structures with sizes in the nanometer range, consistent with vesicular morphology (Figure 2a). Similar vesicular structures were also observed by conventional TEM imaging, further supporting the presence of membrane-bound particles (Figure 2b).

The size distribution of the isolated particles was further characterized by nanoparticle tracking analysis (NTA). The particles exhibited a nanoscale size distribution with a mean diameter of 130.3 nm and a median diameter (×50) of 114.9 nm (Figure 2c). The measured particle concentration was approximately 3.2 × 10^10^ particles/mL, indicating efficient recovery and concentration of vesicular particles from the culture supernatant. The majority of detected particles fell within the typical extracellular vesicle size range, supporting successful enrichment of nano-sized membrane-bound structures.

Total protein content of the EV preparation was quantified using a bicinchoninic acid (BCA) assay and measured at 0.4 mg/mL (Appendix A.2). Based on these measurements, the particle-to-protein ratio was calculated to be approximately 8.0 × 10^10^ particles/mg (8.0 × 10^7^ particles/μg). This relatively high particle-to-protein ratio suggests effective reduction of soluble protein contaminants during processing. The integration of charge-based retention via ExoFilter with downstream TFF appears to enable concurrent enrichment and purification, resulting in EV-enriched preparations characterized by both high particle yield and comparatively low levels of non-vesicular protein components.

To further evaluate surface physicochemical properties, zeta potential measurements were performed. The isolated particles exhibited a negative surface charge, with an average zeta potential of −20.4 mV and a distribution ranging from approximately −44 to −10 mV (Figure 2d). This negative surface charge is consistent with membrane-derived vesicular structures. Importantly, the observed zeta potential aligns with the electrokinetic capture principle underlying the ExoFilter system (Figure 1b,c), in which charge-mediated interactions between negatively charged vesicles and the functionalized filter matrix facilitate selective retention during processing. These findings provide mechanistic support for the effectiveness of the charge-based enrichment strategy.

Although the present study employed ExoFilter-based electrokinetic capture prior to TFF, the workflow is not limited to this sequence. Depending on sample volume and processing requirements, an initial TFF step may be used to reduce bulk volume, followed by ExoFilter-mediated enrichment and a subsequent TFF step for final concentration. Such modular integration is consistent with our previously reported TFF-based concentration strategy [12], which demonstrated efficient nanoscale vesicle recovery under controlled shear conditions. Accordingly, the proposed system should be regarded as an adaptable and modular platform rather than a fixed linear process, allowing optimization according to upstream sample characteristics and downstream application needs.

Collectively, these results demonstrate that the integrated isolation workflow—combining electrokinetic capture with membrane-based filtration—enables recovery of nano-sized vesicular particles exhibiting size distribution and surface charge characteristics consistent with extracellular vesicles. The observed particle yield, particle-to-protein ratio, and surface charge profile support the utility of this approach for generating EV-enriched preparations suitable for subsequent biological and functional analyses.

### 2.2. Effect of Yeast EVs on Dermal Papilla Cell Viability and Metabolic Activity

**Figure 3:**
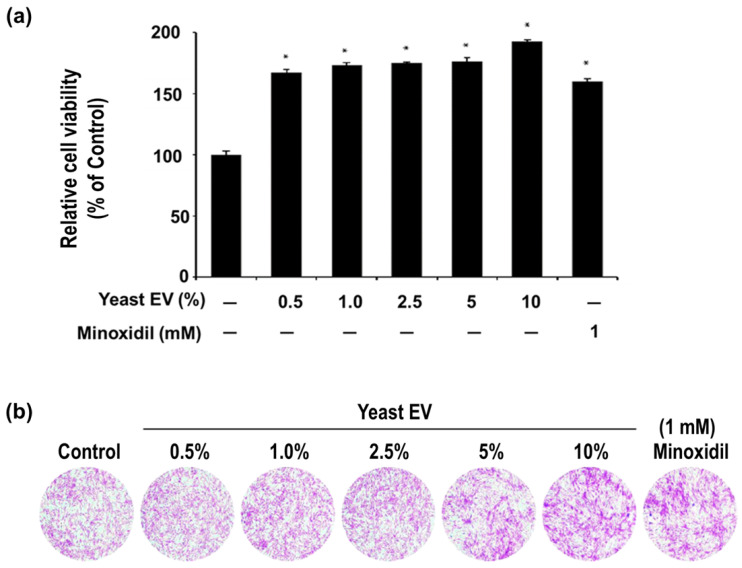
Effects of yeast EVs on cell viability (WST-1 assay) and cell density in human hair follicle dermal papilla cells (hHFDPCs). (**a**) WST-1 assay showing increased cell viability following treatment with increasing concentrations of yeast EVs compared with the untreated control and minoxidil-treated group. (**b**) Crystal violet staining demonstrating increased cell density in EV-treated groups without apparent morphological abnormalities. EV concentrations are expressed as % (*v*/*v*) relative to the culture medium; corresponding particle concentrations are provided in Section 4. Data are presented as mean ± SD (*n* = 3). * indicates *p* < 0.05 vs. control.

The biological activity of yeast EVs was evaluated by assessing their effect on the viability and metabolic activity of human hair follicle dermal papilla cells (hHFDPCs). Treatment with yeast EVs at concentrations ranging from 0.5% to 10% (*v*/*v*), corresponding to approximately 1.6 × 10^8^ to 3.2 × 10^9^ particles/mL, resulted in a statistically significant increase in relative cell viability compared with the untreated control (Figure 3a). Across the tested range, cell viability increased from approximately 165–175% at lower concentrations (0.5–5%) to nearly 190% at 10%, indicating a strong enhancement of cellular metabolic activity.

A concentration-dependent trend was observed, with progressive enhancement of cell viability as the EV dose increased. The maximal effect at 10% EV treatment exceeded that of the positive control, minoxidil (1 mM), which induced an approximate 160% increase relative to control. This finding suggests that yeast EVs exert a potent stimulatory effect on dermal papilla cell activity, comparable to or greater than that of a clinically established hair growth-promoting agent.

Importantly, no reduction in viability was observed at any tested concentration, indicating that the EV preparation did not exert cytotoxic effects within the evaluated range, as defined by cell viability above 80% of the control and absence of morphological abnormalities. Rather, the substantial elevation in metabolic activity and cell number suggests that yeast EVs enhance cellular activity and may support survival-related signaling in dermal papilla cells.

Consistent with the quantitative assay results, crystal violet staining demonstrated increased cell density and coverage in EV-treated groups compared with the untreated control (Figure 3b). The intensity of staining and the apparent confluence of cell layers increased progressively with EV concentration, supporting increased cell accumulation rather than a transient metabolic alteration alone.

Collectively, these findings demonstrate that yeast EVs significantly enhance dermal papilla cell viability and metabolic activity in vitro, with a clear dose-responsive profile and efficacy comparable to a reference hair growth stimulant. These results provide functional evidence supporting the potential application of yeast EVs in promoting hair follicle-associated cellular activity.

### 2.3. Effect of Yeast EVs on Keratinocyte Wound Closure

**Figure 4:**
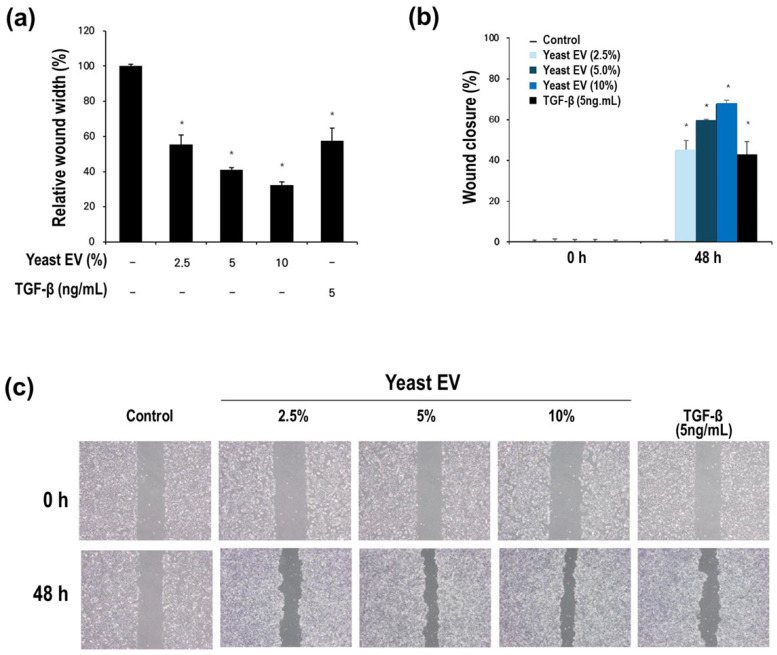
Yeast EVs promote keratinocyte wound closure in HaCaT cells. (**a**) Relative wound width following treatment with yeast EVs at indicated concentrations, with or without TGF-β (5 ng/mL). EV-treated groups showed reduced wound width compared with control. (**b**) Quantitative analysis of wound closure at 48 h demonstrating enhanced migration activity in EV-treated cells. (**c**) Representative scratch assay images at 0 h and 48 h illustrating accelerated wound closure in the presence of yeast EVs in a dose-dependent manner. Data are presented as mean ± SD (*n* = 3). * indicates *p* < 0.05 vs. control.

The effect of yeast-derived EVs on keratinocyte wound closure was evaluated using a scratch wound healing assay in HaCaT cells. Compared with the untreated control, yeast EV treatment markedly accelerated wound closure (Figure 4a,b), suggesting enhanced cell movement associated with wound closure rather than migration alone.

Quantitative analysis demonstrated that EV-treated groups exhibited a substantial reduction in relative wound width after 48 h. While the control group maintained nearly 100% of the initial wound width, EV-treated groups showed a progressive decrease, reaching approximately 40–30% residual wound width at 5–10% EV concentrations (Figure 4a). Correspondingly, wound closure rates increased in a concentration-dependent manner, with 10% EV treatment achieving nearly 65–70% closure at 48 h (Figure 4b). This effect was comparable to—and, at higher EV concentrations, slightly exceeded—that observed with the positive control TGF-β (5 ng/mL), a well-established inducer of keratinocyte wound closure-associated cellular responses.

Importantly, the enhanced wound closure was not attributable to cytotoxic stress-induced contraction, as no morphological abnormalities or detachment were observed in EV-treated cultures. These results suggest active cellular responses contributing to wound closure. Given that dermal papilla cell viability and metabolic activity were also enhanced by yeast EVs (Section 2.2), these findings collectively indicate that yeast EVs may simultaneously modulate multiple cellular components relevant to hair follicle physiology.

Microscopic examination at 0 h and 48 h further supported the quantitative analysis (Figure 4c). EV-treated groups displayed visibly increased cell infiltration into the scratched area, with more uniform and continuous epithelial coverage compared to the control. The progressive narrowing of the wound gap across increasing EV concentrations reinforces the presence of a dose-responsive wound closure effect.

Taken together, these results demonstrate that yeast EVs significantly enhance keratinocyte wound closure in vitro, with efficacy comparable to TGF-β. This effect likely reflects combined contributions from multiple cellular processes, including cell movement and potential proliferation. This enhanced wound closure response suggests a potential role for yeast EVs in facilitating epithelial remodeling and regeneration processes, which are essential components of hair follicle activation and scalp tissue homeostasis.

### 2.4. Anti-Inflammatory Effect of Yeast EVs

**Figure 5:**
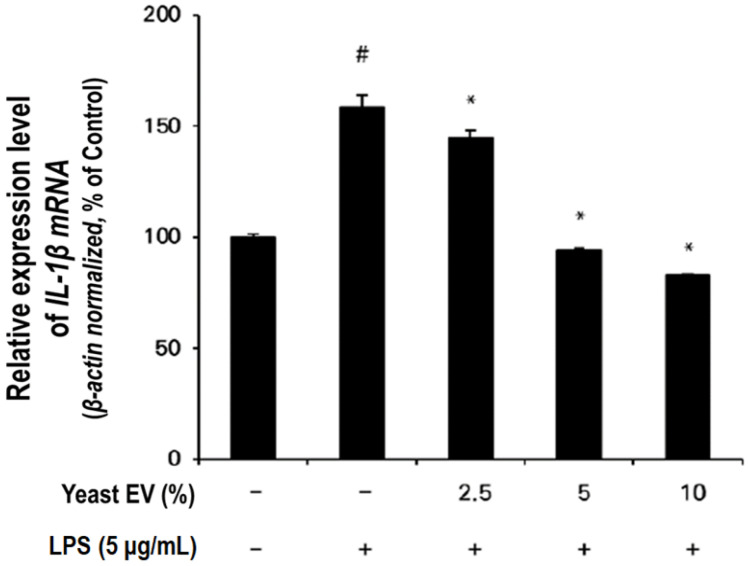
Yeast EVs attenuate inflammatory cytokine expression in LPS-stimulated HaCaT cells. Relative IL-1β gene expression was assessed after LPS stimulation (5 μg/mL) in the presence of yeast EVs at indicated concentrations. LPS markedly increased IL-1β expression, while EV treatment significantly suppressed cytokine levels compared with the LPS group, indicating an anti-inflammatory effect of yeast EVs in keratinocytes. Data are shown as mean ± SD (*n* = 3). * indicates *p* < 0.05, # indicates *p* < 0.01 vs. control.

The anti-inflammatory activity of yeast EVs was investigated by assessing interleukin-1β (IL-1β) mRNA expression in lipopolysaccharide (LPS)-stimulated HaCaT keratinocytes. Exposure to LPS (5 μg/mL) markedly elevated IL-1β expression to approximately 160% of the untreated control level, confirming robust induction of a pro-inflammatory response (Figure 5).

Treatment with yeast EVs significantly attenuated LPS-induced IL-1β upregulation in a concentration-dependent manner. At 2.5% EV treatment, IL-1β expression was reduced to approximately 145% of control, whereas higher concentrations (5% and 10%) further decreased expression to near-baseline levels (approximately 95% and 80–85% of control, respectively). Notably, 10% EV treatment suppressed IL-1β expression below the basal level of the untreated control, suggesting a pronounced modulatory effect on inflammatory signaling.

These results indicate that yeast EVs not only counteract LPS-induced inflammatory activation but may also actively regulate cytokine expression in keratinocytes. The observed dose-responsive suppression supports a biologically meaningful anti-inflammatory effect rather than a nonspecific cytotoxic artifact.

Given that IL-1β plays a central role in initiating epidermal inflammatory cascades and can negatively influence hair follicle cycling under chronic inflammatory conditions, the ability of yeast EVs to modulate IL-1β expression suggests potential utility in maintaining scalp homeostasis. When considered together with the proliferative and migratory effects observed in dermal papilla cells and keratinocytes (Section 2.2 and Section 2.3), these findings suggest that yeast EVs may exert a multifaceted regulatory influence on skin and hair follicle-associated cellular functions.

### 2.5. Effect of Yeast EVs on Collagen Synthesis in Fibroblasts

**Figure 6:**
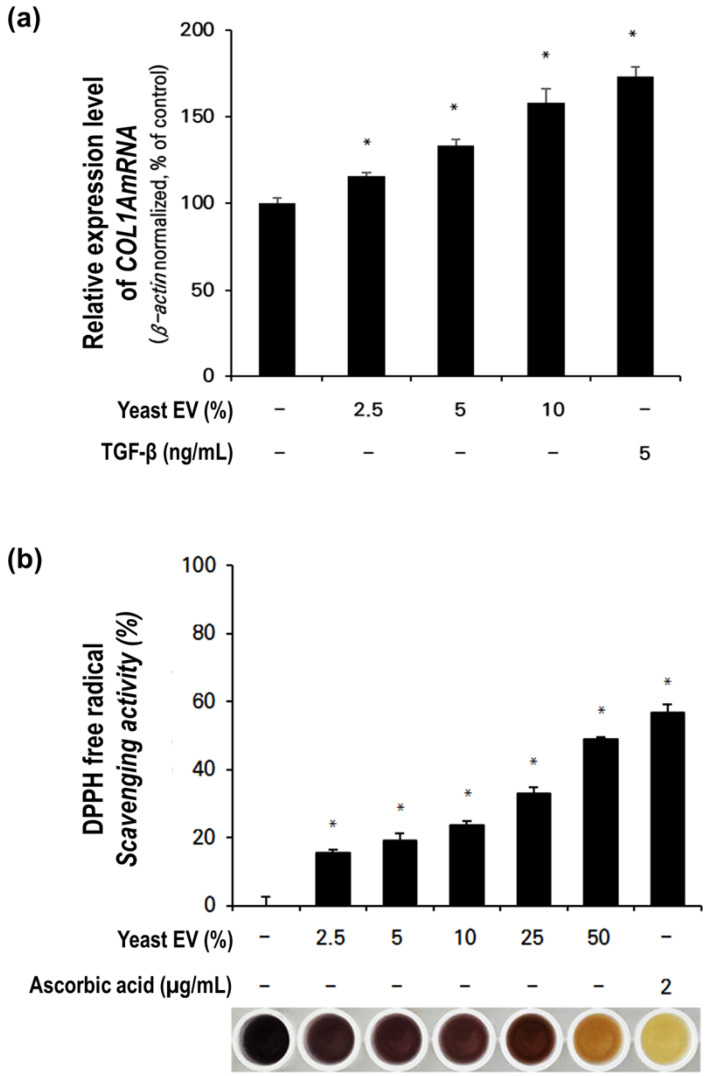
Effects of yeast EVs on dermal collagen production and antioxidant capacity. (**a**) Relative COL1A1 gene expression in NHDF cells following treatment with yeast EVs at indicated concentrations. TGF-β (5 ng/mL) served as a positive control for collagen induction. EV treatment significantly enhanced collagen expression compared with untreated cells. (**b**) DPPH radical scavenging assay demonstrating the antioxidant activity of yeast EVs. Radical scavenging increased with EV concentration, and ascorbic acid (2 μg/mL) was included as a reference control. Representative reaction images are shown below the graph. Data are presented as mean ± SD (*n* = 3). * indicates *p* < 0.05 vs. control.

To investigate whether yeast EVs influence extracellular matrix production, collagen synthesis-related signaling was assessed by measuring COL1A1 mRNA expression in normal human dermal fibroblasts (NHDFs). Treatment with yeast EVs significantly increased COL1A1 expression compared with the untreated control (Figure 6a).

COL1A1 expression progressively increased with EV concentration, rising from approximately 115% at 2.5% EV treatment to nearly 160–170% at 10%. This dose-responsive enhancement indicates active stimulation of collagen-associated transcriptional regulation rather than a transient or nonspecific cellular response. The magnitude of COL1A1 upregulation at higher EV concentrations was comparable to that observed in the positive control group treated with TGF-β (5 ng/mL), a well-established inducer of collagen synthesis in fibroblasts.

Importantly, no morphological abnormalities or reductions in cell viability were observed under the tested conditions, suggesting that the increased COL1A1 expression reflects genuine activation of extracellular matrix-related signaling pathways. These findings indicate that yeast EVs can stimulate collagen-associated gene expression in dermal fibroblasts, supporting a potential role in extracellular matrix remodeling and maintenance of dermal structural integrity.

### 2.6. Antioxidant Activity of Yeast-EVs

The antioxidant potential of yeast EVs was evaluated using a DPPH free radical scavenging assay. Yeast EV treatment resulted in measurable and concentration-dependent radical scavenging activity (Figure 6b). Scavenging capacity increased progressively across the tested range, from approximately 15–20% at lower concentrations (2.5–5%) to nearly 50–60% at the highest concentration tested.

Although the radical scavenging activity of yeast EVs was lower than that of the positive control, ascorbic acid (2 μg/mL), the observed dose-dependent activity indicates that yeast EV preparations possess intrinsic antioxidant capacity. The gradual increase in scavenging efficiency suggests that bioactive components associated with EV membranes or luminal cargo may contribute to redox modulation.

Given that oxidative stress is closely associated with skin aging, inflammation, and hair follicle dysfunction, the antioxidant activity of yeast EVs may provide an additional protective mechanism supporting cellular homeostasis. When considered alongside their proliferative, migratory, anti-inflammatory, and collagen-regulatory effects, these findings further support a multifactorial functional profile of yeast EVs in skin-related cellular systems. Taken together, yeast EVs showed consistent dose-responsive bioactivity across multiple assays, supporting their suitability for downstream mechanistic discussion.

## 3. Discussion

In this study, nano-sized vesicular particles were isolated from Saccharomyces cerevisiae using an integrated electrokinetic-assisted workflow and characterized through complementary physicochemical and functional analyses. Although the classification of fungal vesicles remains under discussion due to the lack of universally conserved mammalian EV markers and biogenesis pathways [3,4,5,6], the isolated particles exhibited hallmark EV-associated features, including membrane-bound morphology, nanoscale size distribution, and a negatively charged surface. These characteristics are consistent with previously described fungal vesicle populations [3,4,5], supporting their identification as biologically relevant vesicular structures.

A key finding of this study is the suggestion of functional activity across multiple skin- and hair follicle-associated cell types. Yeast EV treatment enhanced dermal papilla cell viability and metabolic activity, promoted keratinocyte migration and wound closure, increased fibroblast collagen-related gene expression, attenuated LPS-induced IL-1β expression, and exhibited measurable antioxidant activity. While each effect independently indicates bioactivity, their collective pattern suggests a coordinated and multifunctional mode of action.

We therefore propose that yeast-derived EVs may modulate multiple interconnected processes within the skin and hair follicle microenvironment. Hair follicle function depends on dynamic interactions among dermal papilla cells, keratinocytes, fibroblasts, extracellular matrix components, and local inflammatory mediators [13]. Rather than acting through a single dominant signaling pathway, yeast EVs may influence cellular metabolic activity, migratory, inflammatory, extracellular matrix-related, and redox-regulatory mechanisms in parallel.

The enhancement of dermal papilla cell viability and metabolic activity, together with increased keratinocyte migration and collagen-associated gene expression, suggests modulation of the hair follicle-associated cellular regulation processes involved in follicular microenvironment maintenance [14]. However, it should be noted that wound closure over extended incubation periods may be influenced by both cell migration and proliferation. Therefore, the present results are interpreted as enhanced wound closure rather than migration alone. Simultaneous suppression of pro-inflammatory cytokine expression and the presence of antioxidant activity indicate attenuation of inflammatory and oxidative stress-associated signaling. Chronic low-grade inflammation and oxidative imbalance are recognized contributors to hair follicle dysfunction and tissue aging [15,16]. Accordingly, the dual pro-regenerative and protective activities observed here may contribute to a microenvironment that is more favorable for tissue homeostasis.

Extracellular vesicles are increasingly recognized as multifunctional signaling entities capable of transferring diverse molecular cargo and modulating multiple recipient cell pathways simultaneously [17]. The coordinated responses observed across different skin-related cell types are consistent with a microenvironment-level modulation model, in which yeast EVs act as bioactive particulate mediators rather than single-pathway stimulators.

From a methodological perspective, isolation of vesicles from dense microbial suspensions presents challenges distinct from mammalian systems. Conventional approaches, including ultracentrifugation and size-based filtration, may encounter limitations related to clogging, heterogeneity, or scalability [18]. The integrated workflow employed here—combining electrokinetic capture with subsequent filtration and concentration—was designed to address these constraints. The charge-based enrichment step [19] enabled selective recovery of negatively charged vesicular structures while reducing loosely associated impurities, thereby improving processing efficiency. This workflow may provide a scalable and reproducible alternative to conventional ultracentrifugation-based fungal EV isolation methods.

Comparison with established reference compounds further contextualizes the functional relevance of yeast EVs. Minoxidil promotes follicular cell activity primarily through vascular and signaling mechanisms [11]. In the present study, yeast EVs induced dermal papilla cellular activity comparable to that observed with minoxidil treatment. However, unlike small-molecule agents that typically act through relatively defined pathways, EVs represent biological delivery systems capable of transferring multiple bioactive components simultaneously [17]. Similar multifunctional effects have been reported in regenerative and dermatological contexts [9,10], and the present findings extend this concept to vesicles derived from yeast, a scalable biological source.

Several limitations of the present study should be acknowledged. First, detailed molecular cargo profiling was not performed, and the specific mediators responsible for the observed biological effects remain unidentified. In addition, mechanistic signaling pathways were not directly examined, and all functional assays were conducted in vitro. Therefore, further investigations incorporating comprehensive cargo characterization, pathway-level analyses, and in vivo validation will be required to elucidate the underlying mechanisms and assess the physiological and translational relevance of yeast-derived EVs.

In terms of vesicle characterization, the present study primarily relied on physicochemical properties, including morphology (TEM/cryo-TEM), size distribution (NTA), surface charge (zeta potential), and particle-to-protein ratio. While these features are consistent with vesicular structures, they are not sufficient to definitively establish vesicle identity according to MISEV guidelines. In particular, proteomic characterization was not performed, and therefore, the lack of molecular marker validation limits definitive classification of these particles as extracellular vesicles. In addition, luminal cargo validation experiments, such as RNase or Proteinase K protection assays with or without detergent treatment, were not conducted to confirm the presence of membrane-enclosed luminal cargo. The absence of negative markers further limits the ability to exclude co-isolated non-vesicular components. Accordingly, it remains possible that a fraction of the isolated particles may include non-vesicular nanoparticles that cannot be excluded under the current characterization framework. Future studies incorporating proteomic or multi-omics analyses, membrane protection assays, and the inclusion of both positive and negative markers will be essential to strengthen vesicle validation and purity assessment.

Regarding functional assays, the anti-inflammatory effect was evaluated based on IL-1β mRNA expression. In addition, cell viability assessed by WST-1 assay reflects metabolic activity rather than direct cell proliferation. Therefore, further validation using direct cell counting or proliferation-specific assays (e.g., EdU/BrdU incorporation or Ki-67 staining) will be required to confirm proliferative effects. However, protein-level validation (e.g., ELISA) and broader cytokine profiling will be necessary to provide a more comprehensive understanding of the immunomodulatory effects of yeast-derived EVs. Similarly, while COL1A1 mRNA expression provides an initial indication of collagen-related activity, protein-level validation will be required to confirm functional extracellular matrix remodeling.

In addition, the DPPH assay reflects chemical radical scavenging activity and does not directly represent intracellular antioxidant effects. Therefore, further studies using cell-based reactive oxygen species (ROS) assays will be necessary to validate antioxidant activity under biologically relevant conditions. These limitations highlight important directions for future studies aimed at strengthening the biological, mechanistic, and translational significance of yeast-derived EVs.

In summary, the present findings indicate that yeast-derived vesicular particles exhibit physicochemical characteristics consistent with extracellular vesicles and exert coordinated biological effects across multiple skin-related cell types. The integrated functional profile observed—spanning regenerative, anti-inflammatory, extracellular matrix modulatory, and antioxidant activities—supports a microenvironment-level modulation model. These results suggest that yeast-derived EVs may function as multifunctional biological mediators associated with the modulation of interconnected cellular processes relevant to hair follicle physiology.

## 4. Materials and Methods

### 4.1. Isolation Saccharomyces Cerevisiae-Derived Extracellular Vesicles

Dried beer yeast was purchased from Beer Yeast Korea (Seoul, Korea). For revival, the powder was first rehydrated in sterile distilled water and inoculated into yeast extract–peptone–dextrose (YPD) medium. Cultures were incubated at 30 °C with shaking to allow for cellular recovery and entry into active growth. The revived culture was subsequently expanded in fresh YPD medium and grown to the logarithmic phase. The actively growing culture was then transferred into a larger-scale production culture and further cultivated in YPD medium to a final working volume of 10 L. After reaching the logarithmic growth phase, the culture supernatant was collected following removal of cells and debris and processed for extracellular vesicle isolation.

Processing such a large-volume suspension presents technical limitations for conventional EV isolation approaches commonly used at the laboratory scale, including ultracentrifugation and size-exclusion chromatography. In addition, direct application of tangential flow filtration (TFF) to the crude yeast suspension resulted in rapid pore blockage due to the presence of abundant nano-sized particulate materials, thereby preventing efficient filtration. The integration of charge-based capture with downstream membrane filtration has previously been shown to enhance vesicle recovery and process robustness compared with single-step filtration approaches [12].

To address these limitations, the isolation workflow illustrated in Figure 1a was implemented. Initially, the large-volume yeast suspension was gently agitated at low speed to facilitate the release of vesicular particles from dried yeast material. Because the crude suspension could not be directly subjected to centrifugation, it was allowed to sediment at 4 °C for 24 h. The clarified supernatant was then carefully collected using a pump to avoid disturbing the settled material.

The recovered supernatant was subjected to low-speed centrifugation (800× *g*, 15 min) to remove remaining intact cells and large debris, followed by a second centrifugation step at 3000× *g* to further eliminate residual cellular fragments. The resulting supernatant was subsequently filtered through an 800 nm membrane to achieve additional pre-clarification. Despite these preparatory steps, direct tangential flow filtration (TFF) remained inefficient due to membrane fouling, necessitating the implementation of the electrokinetic capture step described below.

Therefore, an electrokinetic EV capture step was introduced prior to TFF processing. Negatively charged vesicles were selectively retained on a cationic mesh matrix through electrostatic interactions. The captured vesicles were subsequently eluted using an optimized buffer designed to disrupt these interactions, yielding an approximately five-fold enrichment of the EV fraction. The eluate was then subjected to tangential flow filtration for further concentration and buffer exchange. Additional TFF processing was performed to achieve the desired final concentration. The resulting EV preparation was collected under sterile conditions and stored appropriately until use in downstream analyses.

### 4.2. Electrokinetic EV Isolation Using ExoFilter

To facilitate scalable EV isolation from large-volume yeast suspensions, an electrokinetic filtration step was introduced prior to tangential flow filtration (TFF). This step was performed using a proprietary electrokinetic capture system, ExoFilter (60250, Microgentas, Seoul, Korea) developed in our laboratory [19]. The system incorporates a positively charged mesh matrix designed to selectively retain negatively charged vesicular structures from clarified suspensions.

As illustrated in Figure 1b, the yeast suspension (450 mL) was passed through the ExoFilter unit (bottle-top type) under controlled flow conditions (gravity-driven flow, ~1 g), enabling electrostatic interactions between the mesh surface and vesicular particles. The filter consists of a multilayered mesh structure, which provides an increased surface area and sufficient interaction opportunities for vesicular particles to contact positively charged surfaces.

During this electrokinetic filtration process (Figure 1c), negatively charged vesicles are preferentially retained on the mesh, while weakly interacting components and smaller non-vesicular impurities pass through, contributing to reduced background contamination. Subsequently, a mild vacuum pressure (~15 kPa) was further applied to remove residual liquid and loosely associated impurities from the porous mesh structure. Following this washing step, the retained vesicular particles were eluted using a buffer designed to disrupt electrostatic interactions.

Although a high-salt elution buffer (e.g., 1 M NaCl) has been recommended in our previous studies [12,19], such conditions may introduce osmotic stress and potentially affect bioactive components. Therefore, in the present study, phosphate-buffered saline (PBS) was used as a milder elution buffer to preserve the functional integrity of the EVNPs. This elution process is conceptually analogous to ion-exchange-based release.

The recovered eluate was subsequently concentrated and buffer-exchanged using tangential flow filtration (TFF). Additional methodological details can be found in our previous studies [12,19]. The process was reproducible across independent runs, yielding consistent particle size distributions and concentrations.

### 4.3. Physicochemical Characterization of EVs

The morphology of the isolated vesicles was examined using cryogenic transmission electron microscopy (cryo-TEM) and conventional transmission electron microscopy (TEM). For cryo-TEM analysis, EV samples were vitrified on lacey carbon grids and imaged to confirm vesicular structure and membrane integrity. Conventional TEM imaging was performed after negative staining to further verify particle morphology and structural features.

Particle size distribution, particle concentration, and zeta potential of the isolated vesicles were measured using a nanoparticle tracking analysis system (ZetaView-QUATT, Particle Metrix, Wildmoos, Germany). EV samples were diluted in particle-free buffer to achieve optimal particle counts within the recommended detection range prior to measurement. Hydrodynamic diameter and particle concentration were calculated from multiple recorded videos using the instrument software (ZetaSphere software, V.1).

For zeta potential analysis, EV samples were diluted in a low-conductivity buffer and analyzed using the same ZetaView system under electrophoretic measurement mode. The average zeta potential value was obtained from repeated measurements. All measurements were performed at least in triplicate to ensure reproducibility.

### 4.4. Cell Culture

Human hair follicle dermal papilla cells (hHFDPCs), human keratinocyte cells (HaCaT), and normal human dermal fibroblasts (NHDFs) were used in this study. Cells were maintained in their respective culture media, supplemented with fetal bovine serum and antibiotics under standard conditions (37 °C, 5% CO_2_). Cells were subcultured upon reaching appropriate confluence and used within a limited passage range to ensure experimental consistency. For all experiments, cells were seeded at defined densities and allowed to stabilize overnight prior to treatment with extracellular vesicle (EV) samples.

### 4.5. Cell Viability and Metabolic Activity Assay

The proliferative effect of yeast EV on hHFDPCs was evaluated using a WST-1-based cell viability assay. Cells were seeded in 96-well plates at a defined density and incubated for 24 h. After attachment, the medium was replaced with fresh medium containing yeast EVs at the indicated concentrations. Cells were incubated for an additional 48 h.

Following treatment, WST-1 reagent (EZ-CYTOX; DOGEN, Seoul, Korea) was added to each well and incubated at 37 °C for the time recommended by the manufacturer. Absorbance was measured using a microplate reader (SpectraMax^®^ i3x Multi-Mode Detection Platform; Molecular Devices, San Jose, CA, USA) at 450 nm with a reference wavelength of 650 nm. Cell viability was expressed as a percentage relative to the untreated control group. Non-cytotoxic conditions were defined as those in which cell viability remained above 80% of the untreated control, with no observable morphological abnormalities under microscopic examination.

In addition, cell morphology was examined by crystal violet staining. Cells were stained with 0.2% crystal violet solution (Sigma, St. Louis, MO, USA) and observed under an inverted microscope (OLYMPUS, Tokyo, Japan).

Yeast-derived EVs were administered to cells on a volume/volume (*v*/*v*) basis. The stock EV preparation contained 3.2 × 10^10^ particles/mL, as determined by nanoparticle tracking analysis (NTA). Final treatment concentrations corresponded to approximately 1.6 × 10^8^, 3.2 × 10^8^, 8.0 × 10^8^, 1.6 × 10^9^, and 3.2 × 10^9^ particles/mL for 0.5%, 1%, 2.5%, 5%, and 10% EV treatments, respectively.

### 4.6. Wound Healing Assay

Cell migration was assessed using a scratch wound healing assay in HaCaT cells (Gibco, Waltham, MA, USA). Cells were seeded in culture plates and grown to near-confluence. A linear scratch was created using a sterile pipette tip, and detached cells were removed by washing with phosphate-buffered saline (PBS). Cells were subsequently treated with yeast EV at the indicated concentrations and incubated for up to 48 h. Images of the wound area were captured immediately after scratching (0 h) and after incubation using the inverted microscope, along with a camera. Wound closure was quantified by measuring the remaining wound width relative to the initial scratch area.

### 4.7. Inflammatory Cytokine Gene Expression Analysis

The anti-inflammatory effect of yeast EV was evaluated by measuring pro-inflammatory cytokine gene expression using quantitative real-time PCR (qRT-PCR) in HaCaT keratinocytes. HaCaT cells were seeded in 6-well plates at a density of 2 × 10^5^ cells per well and incubated for 24 h. The cells were then treated with yeast EVs at the indicated concentrations and cultured for an additional 24 h under standard conditions.

Total RNA was extracted using TRIzol reagent (Invitrogen, Carlsbad, CA, USA) according to the manufacturer’s protocol. Complementary DNA (cDNA) was synthesized using M-MLV reverse transcriptase (Invitrogen, USA). Quantitative PCR was performed using SYBR™ Green Master Mix (Applied Biosystems, Foster City, CA, USA) on a StepOnePlus™ Real-Time PCR System (Applied Biosystems, USA).

The PCR cycling conditions consisted of an initial denaturation at 94 °C for 12 min, followed by 40 cycles of denaturation at 94 °C for 20 s, annealing at 60 °C for 30 s, and extension at 72 °C for 30 s. Gene expression levels were normalized to β-actin as an internal reference, and relative expression was calculated using the 2^−ΔΔCt^ method. Data were expressed as mean ± standard deviation from three independent experiments. Statistical significance was evaluated using Student’s *t*-test (*p* < 0.05). The primer sequences used in this study are listed in Appendix A (Table A1).

### 4.8. Collagen Gene Expression Analysis

The effect of yeast EV on collagen synthesis was evaluated by measuring COL1A1 mRNA expression using quantitative real-time PCR in normal human dermal fibroblasts (NHDFs). NHDF cells were seeded in 6-well plates at a density of 2 × 10^5^ cells per well and cultured for 24 h. The cells were then treated with yeast EVs at the indicated concentrations, or with control substances, and incubated for an additional 24 h. TGF-β was used as a positive control for collagen induction.

Total RNA was extracted using TRIzol reagent (Invitrogen, USA), and cDNA was synthesized using M-MLV reverse transcriptase (Invitrogen, USA). Quantitative PCR was performed using SYBR™ Green Master Mix on a StepOnePlus™ Real-Time PCR System (Applied Biosystems, USA).

COL1A1 expression levels were normalized to β-actin as an internal reference, and relative expression was calculated using the comparative Ct (2^−ΔΔCt^) method. Data are presented as mean ± standard deviation from three independent experiments. Statistical significance was determined using Student’s *t*-test (*p* < 0.05). Primer sequences are listed in Appendix A (Table A1).

### 4.9. Antioxidant Activity Assay

The antioxidant activity of yeast EVs was evaluated using a DPPH free radical scavenging assay. The EV sample, supplied in liquid form, was diluted with sterile distilled water to the indicated concentrations. Ascorbic acid (Sigma-Aldrich, St. Louis, MO, USA) dissolved in PBS was used as a positive control.

A 0.2 mM DPPH solution prepared in methanol was mixed with the test sample and incubated in the dark at room temperature for 30 min. Absorbance was measured at 517 nm using a microplate reader (SpectraMax^®^ i3x Multi-Mode Detection Platform, Molecular Devices, USA). Radical scavenging activity was calculated using the standard formula based on sample and control absorbance values.

### 4.10. Statistical Analysis

Statistical analyses were performed using one-way analysis of variance (ANOVA), followed by post hoc multiple comparison tests (e.g., Tukey’s test). All experiments were performed in triplicate (*n* = 3, biological replicates), unless otherwise stated. Data are presented as mean ± standard deviation (SD).

## 5. Conclusions

This study establishes an electrokinetic-assisted strategy for isolating nano-sized vesicular particles from Saccharomyces cerevisiae suitable for large-volume processing. The recovered particles display extracellular vesicle-like physicochemical properties and demonstrate measurable bioactivity in diverse skin-relevant cellular assays. Beyond individual functional effects, the data collectively suggest that yeast-derived EVs represent a scalable bioactive platform with potential applications in skin and hair biology. Future studies focusing on molecular cargo characterization, mechanistic pathway analysis, and in vivo validation will be critical for defining their translational value in biomedical and cosmetic contexts.

## Figures and Tables

**Figure A1:**
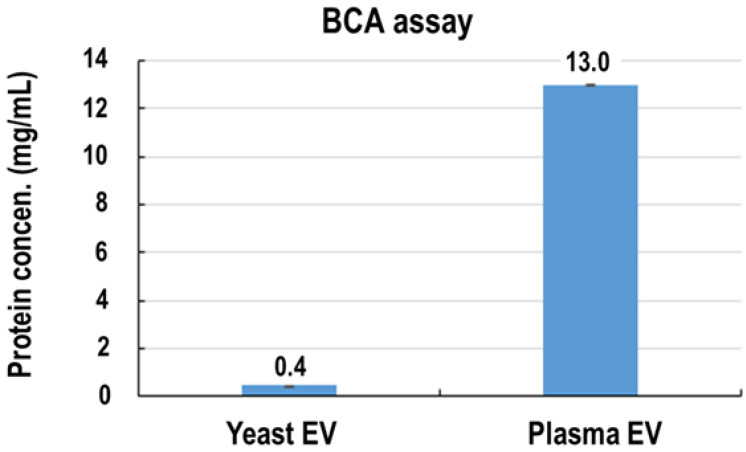
BCA-based protein quantification of EV preparations. Protein concentration of yeast-derived extracellular vesicle (EV) preparations was quantified using a bicinchoninic acid (BCA) assay. Yeast EVs exhibited a protein concentration of 0.4 mg/mL. For comparison, plasma-derived EV preparations showed a protein concentration of 13.0 mg/mL under similar measurement conditions. Measurements were performed in independent replicates.

## Data Availability

The main data supporting the results of this study are available within the manuscript. The raw data files are available for research purposes from the corresponding author upon reasonable request. Source data are provided with this paper.

## Abbreviations

EV	Extracellular vesicle
SC	Saccharomyces cerevisiae
hHFDPCs	Human hair follicle dermal papilla cells

## Appendix A

### Appendix A.1

**Table A1:** Primer information for qRT-PCR analysis of antioxidant assay.

Gene	Forward Primer (5′ → 3′)	Reverse Primer (5′ → 3′)
IL-1β	CCACAGACCTTCCAGGAGAATG	GTGCAGTTCAGTGATCGTACAGG
β-actin	GGATTCCTATGTGGGCGACGA	CGCTCGGTGAGGATCTTCATG

**Table A2:** Primer information for qRT-PCR analysis of collagen assay.

Gene	Forward Primer (5′ → 3′)	Reverse Primer (5′ → 3′)
COL1A1	AGGGCCAAGACGAAGACATC	AGATCACGTCATCGCACAACA
β-actin	GGATTCCTATGTGGGCGACGA	CGCTCGGTGAGGATCTTCATG
